# Mechanistic Insights into Polyphenol Oxidase Inhibition by Four Bioactive Constituents from *Hydrangea macrophylla* Serrata: Antioxidant Activity of Single Compounds Compared to the Whole-Leaf Extract

**DOI:** 10.3390/molecules31193411

**Published:** 2026-09-24

**Authors:** Sijie Mei, Thomas Heymann, Volker Böhm

**Affiliations:** 1 Institute of Nutritional Sciences, Friedrich Schiller University Jena, 07743 Jena, Germany; sijie.mei@uni-jena.de; 2 Institute of Chemistry—Food Chemistry, Martin-Luther-University Halle-Wittenberg, 06120 Halle (Saale), Germany; thomas.heymann@chemie.uni-halle.de

**Keywords:** *Hydrangea macrophylla*, chlorogenic acid, hydrangenol, hydrangeic acid, phyllodulcin, antioxidant activity, anti-browning activity, molecular docking

## Abstract

*Hydrangea macrophylla* serrata (tea-hortensia) leaves are traditionally used to produce a sweet herbal tea (“Amacha”) and contain hydroxycinnamates and dihydroflavonols with potential bioactivities (e.g., anti-inflammatory, anti-allergy). In this study, from an extract of tea-hortensia leaves, we isolated and structurally characterized four major constituents: chlorogenic acid (CGA, 5-caffeoylquinic acid), hydrangenol (HG), hydrangeic acid (HGA), and phyllodulcin (PD). The four constituents and the extract were evaluated for antioxidant capacity via total phenolic content (TPC), ABTS radical scavenging (TEAC: Trolox equivalent antioxidant capacity), and oxygen radical absorbance capacity (ORAC) assays, and for anti-browning potential via polyphenol oxidase (PPO) inhibition assays. To elucidate the underlying mechanisms, we employed kinetic analysis to determine inhibition types, evaluated copper-chelating capacity, and performed molecular docking simulations to investigate ligand–PPO binding modes and key interacting residues. The tea extract exhibited significantly superior antioxidant capacity compared to all isolated compounds across all assays, with CGA being the most potent among the four compounds. For anti-browning activity, CGA and PD showed the strongest PPO inhibition (90%), while the tea extract (83% at 2 mg/mL) outperformed HG and HGA but was less potent than CGA and PD. Molecular docking further revealed that CGA formed stable interactions with key residues in the PPO active site. These findings provide a systematic comparative assessment of the anti-browning mechanisms of the major constituents of *H. macrophylla* serrata, highlighting the potential of this plant as a natural source of antioxidants and anti-browning agents for food applications. The integration of biochemical kinetics, copper-chelating analysis, and in silico docking offers a comprehensive mechanistic framework for understanding PPO inhibition by natural phenolic compounds.

## 1. Introduction

*Hydrangea macrophylla* serrata (family Hydrangeaceae), commonly known as tea-hortensia, is a deciduous shrub native to Japan and Korea. The processed leaves of this plant are traditionally used for “Amacha,” a sweet Japanese herbal tea whose characteristic sweetness derives from phyllodulcin, a dihydroisocoumarin approximately 400–800 times sweeter than sucrose [1]. Beyond phyllodulcin, previous phytochemical analyses have revealed that *H. macrophylla* serrata leaves contain a diverse array of secondary metabolites, including dihydroisocoumarins (hydrangenol and its derivatives), dihydrostilbenoids (hydrangeic acid), flavonoids (kaempferol and quercetin glycosides), cyanogenic glucosides (hydrangin), and cinnamic acid derivatives (chlorogenic acid) [2,3,4,5]. This structural diversity directly underpins the potential applications of the plant’s bioactive natural products in the food and pharmaceutical industries.

Among the various constituents present in *H. macrophylla* subsp. serrata leaves, four compounds representing distinct structural classes have garnered particular attention for their bioactivities. Chlorogenic acid (CGA), a well-characterized phenolic acid commonly found in many plant species, has been extensively documented for its antioxidant properties and its ability to inhibit polyphenol oxidase (PPO), a key enzyme responsible for enzymatic browning in fruits and vegetables [6]. Hydrangenol (HG), a dihydroisocoumarin, has been reported to exhibit potent antioxidant activity, with antioxidant and anti-browning effects through PPO inhibition [1]. Phyllodulcin (PD), another dihydroisocoumarin, has demonstrated antioxidant capacity through the enhancement of catalase activity and scavenging of reactive oxygen species [7]. In contrast, hydrangeic acid (HGA), a dihydrostilbenoid structurally distinct from the dihydroisocoumarins, has been primarily investigated for its anti-diabetic and anti-inflammatory properties, with limited information available on its antioxidant and anti-browning activities [5].

PPO inhibition mechanisms generally fall into several categories, including copper chelation at the active site, competitive binding by substrate analogs, mixed-type/noncompetitive inhibition, and redox-mediated prevention of quinone polymerization [8]. The PPO inhibition rate of HG was reported by Al-Yafei [1]. However, systematic investigations into the anti-browning mechanisms of hydrangenol, hydrangeic acid, and phyllodulcin remain unexplored. Moreover, the comparative efficacies of these four structurally distinct compounds and their relationship to whole-leaf extract activity have not been established. The aim of our study is to provide a comprehensive comparative assessment of these four constituents, with particular emphasis on the mechanistic basis of their PPO inhibition, and a comparison of the single compounds with the whole-leaf extract to clarify whether the overall bioactivity is dominated by a single constituent or arises from synergistic interactions.

## 2. Results and Discussion

### 2.1. Identification and Quantification

#### 2.1.1. HPLC-DAD

Chlorogenic acid, hydrangenol, and phyllodulcin were identified by comparing their retention times with those of authentic reference standards using HPLC-DAD.

#### 2.1.2. LC-MS-MS

Hydrangeic acid (HGA) was identified via LC-MS-MS analyses. Primarily, the corresponding pseudo-molecular ions [M + H]^+^ of 256.9 *m*/*z* and [M + Na]^+^ of 279.3 *m*/*z* were detected in full scan mode (MS1) for a peak at 26.30 min. Subsequently, a collision-induced dissociation (CID) of the protonated molecule [M + H]^+^ led to specific fragment ions of 211.2 *m*/*z* (-CO_2_), 193.2 *m*/*z* (-CO_2_, -H_2_O) and 165.2 *m*/*z* (-phenol), proving the presence of hydrangeic acid (HGA). The LC-MS-MS spectra are provided as Figures S1 and S2 in the Supplementary Material.

### 2.2. Antioxidant Activities

#### 2.2.1. Antioxidant Activities of Four Compounds

The antioxidant activities of the four isolated compounds were evaluated via their total phenolics content (TPC), Trolox equivalent antioxidant capacity (TEAC), and oxygen radical absorbance capacity (ORAC) (Table 1). CGA exhibited the highest TPC value (3.69 g GAE/100 g), significantly surpassing the other compounds, followed by PD (1.77), HGA (1.12), and HG (0.75), with all compounds showing significant differences from each other (*p* < 0.05). In the TEAC assay, CGA again showed the highest capacity (0.20 mM TE/mM), forming a statistically comparable group (0.13–0.20 mM TE/mM) with HGA and PG, while HG displayed a significantly lower value (0.08 mM TE/mM). For ORAC, CGA remained the most potent (2.45 mM TE/mM), PD (2.18 mM TE/mM) and HGA (2.00 mM TE/mM) were intermediate, and HG (1.41 mM TE/mM) was the weakest. In all assays, antioxidant potency ranked CGA > PD > HGA > HG, with CGA strongest and HG weakest.

#### 2.2.2. Comparing the Antioxidant Activities of Four Compounds and Tea Extract

The tea extract exhibited significantly higher antioxidant capacity across all three assays compared to all isolated compounds (*p* < 0.05; Table 2). In the TPC assay, the tea extract (3.95 g GAE/100 g) showed the highest phenolics content, slightly but significantly exceeding that of CGA (3.69 g GAE/100 g). In both the TEAC and ORAC assays, the tea extract demonstrated approximately twice the antioxidant capacity of the most potent isolated compound (CGA), with TEAC values of 1.09 vs. 0.56 mmol/g and ORAC values of 12.26 vs. 6.92 mmol/g, respectively. These results indicate the superior antioxidant properties of the tea extract, likely accounted for by combined effects among its multiple phenolic constituents.

### 2.3. Polyphenol Oxidase Inhibitory Activity

#### 2.3.1. Inhibition Rate

All compounds exhibited clear concentration-dependent inhibitory activity, with inhibition rates increasing as the concentration rose from 0.125 to 2 mg/mL (Figure 1). Chlorogenic acid (CGA) and phyllodulcin (PD) showed nearly identical inhibition curves, reaching 90% inhibition at the highest tested concentration, while hydrangenol (HG) and hydrangeic acid (HGA) displayed similar but lower capacity, with maximum inhibition rates of approximately 70%. The inhibition rate at 2 mg/mL was in the order CGA (91%) = PD (90%) > HGA (70%) = HG (68%). The IC_50_ value of the whole-leaf extract was determined to be 0.45 mg/mL, which was lower than that of HG (0.70 mg/mL) but higher than those of HGA (0.44 mg/mL), CGA (0.43 mg/mL) and PD (0.35 mg/mL). This suggests either that the whole tea extract contains more effective anti-browning compounds or that the interactions between the compounds also contribute to its activity.

#### 2.3.2. Inhibition Kinetics

Kinetic analysis revealed distinct inhibition mechanisms of the tested compounds against PPO (Table 3). As a known PPO substrate, chlorogenic acid (CGA) exhibited mixed-type inhibition (Kis = 0.080 mM, Kii = 1.47 mM), which is consistent with previous findings that CGA inhibits PPO in a reversible mixed-type manner [6]. This suggests that CGA interacts with both the free enzyme and the enzyme–substrate complex.

Notably, HG and HGA demonstrated competitive inhibition, with Kis values of 0.147 mM and 0.131 mM, respectively, while their Kii values were substantially larger (8.20 mM and 7.28 mM). The near-unaffected Vmax values further support that these compounds primarily compete with the substrate for the active site, which may be attributed to its structural similarity to phenolic substrates. Phyllodulcin (PD) showed mixed-type inhibition (Kis = 0.184 mM, Kii = 2.71 mM), indicating interactions with both the free enzyme and the enzyme–substrate complex, possibly involving binding near the dinuclear copper active center as described for other inhibitors. These findings highlight Hydrangea-derived compounds, particularly HG and HGA, as promising competitive PPO inhibitors for potential applications in controlling enzymatic browning.

#### 2.3.3. Recovery Rate

The effect of copper ion addition on PPO activity recovery after inhibitor pretreatment is shown in Figure 2. At both 0.125 mg/mL and 0.25 mg/mL concentrations, HG exhibited recovery rates of 48%, while HGA showed 47% and 65%, and PD displayed 68% and 80%. In contrast, CGA demonstrated consistently low recovery rates of 14% at both concentrations, and the tea extract showed 6% and 7%, respectively.

The substantial restoration of PPO activity after Cu^2+^ addition indicates that PD, HGA and HG inhibit PPO primarily through reversible copper chelation at the enzyme’s active site. PPO is a type-3 copper metalloenzyme containing two copper ions (CuA and CuB) coordinated by histidine residues. When chelating inhibitors bind to these copper ions, they disrupt the enzyme’s catalytic cycle. The subsequent addition of exogenous Cu^2+^ competes with the inhibitor for the metal-binding site, effectively displacing the inhibitor and restoring enzymatic activity. The high recovery rates observed for PD, HGA and HG suggest that their binding to copper is reversible and that the apoenzyme (copper-depleted PPO) can be efficiently reconstituted upon copper addition. The partial restoration of PPO activity after Cu^2+^ addition is consistent with the involvement of reversible copper interactions. However, this result alone cannot distinguish among direct copper chelation, displacement of the inhibitor from the active site, and reconstitution of the apoenzyme. These mechanisms therefore remain possible interpretations rather than established conclusions.

The minimal activity recovery of CGA and tea extract after Cu^2+^ addition suggests that CGA and the tea extract inhibit PPO through non-chelating mechanisms. CGA may act as an inhibitor by competing with phenolic substrates for the active site binding pocket and interacting with the copper ions themselves. For the tea extract, the low recovery rate likely reflects the combined effects of multiple constituents—while individual components may chelate copper, other compounds in the extract may inhibit PPO through non-chelating pathways or cause irreversible conformational changes in the enzyme, rendering the active site inaccessible even after copper is replenished.

### 2.4. Correlation

Spearman’s correlation coefficients were calculated among six key parameters: inhibition rate, recovery rate, ${IC}_{50}$, TEAC, TPC, and ORAC (Figure 3). For TPC, a strong positive correlation was found with ORAC (r = 1), as well as a moderate positive correlation with TEAC (r = 0.7), suggesting that phenolic compounds are major contributors to the observed antioxidant activities. Inhibition rate showed a strong negative correlation with ${IC}_{50}$ (r = −0.9) and moderate correlations with ORAC (r = 0.7) and TPC (r = 0.7), as well as weak correlation with TEAC (r = 0.2). Moderate negative correlations were further noted between recovery rate and TEAC (r = −0.5) and between ${IC}_{50}$ and TPC (r = −0.6).

Some studies showed that phenolic compounds act as efficient electron donors, reducing the highly reactive o-quinones, the primary products of polyphenol oxidase (PPO)-catalyzed oxidation, back to their corresponding diphenols via single-electron transfer, thereby blocking the polymerization cascade that generates brown pigments [9,10]. Additionally, phenolic compounds can coordinate with the binuclear copper center at the PPO active site, which can directly attenuate enzymatic activity through metal chelation [11]. This dual mechanism of electron donation to quinones and copper coordination explains why phenolic-rich samples exhibit both high antioxidant activity and strong PPO inhibition and recovery. Notably, since the correlations were calculated from only four compounds, they should be regarded as descriptive statistics for the present observations and not generalized to other compounds.

### 2.5. Molecular Docking

A molecular docking method was used to explore the mode of interactions of these four compounds with PPO. The binding energy values between PPO and CGA, HG, HGA, and PD were −8.3 kcal mol^−1^, −7.4 kcal mol^−1^, −7.6 kcal mol^−1^ and −7.6 kcal mol^−1^, respectively. It has been reported that the affinity of the ligand to the receptor protein is negatively correlated with its binding energy [12]. Our results indicated that all four of these compounds could spontaneously bind to PPO, and CGA is most stable in PPO binding. Molecular docking analysis (Figure 4) revealed that the interactions between chlorogenic acid and the PPO receptor are predominantly governed by hydrogen bonding with TRP244, ASP178, and ASP186, alongside van der Waals contacts involving ASN187, THR175, THR184, HIS171, TYR181, LEU176, VAL177, THR193, VAL188, and ASN170, while GLU185 participates in a Pi-anion interaction. For hydrangeic acid, hydrogen bonds are formed with VAL188 and HIS171; hydrophobic interactions are established with LYS190 and LEU176; van der Waals forces involve SER189, GLU185, THR184, TYR181, TRP244, and ASP178; and a Pi-anion interaction is observed with ASP186. In the case of hydrangenol, hydrogen bonding occurs with ASP186 and ASP178; hydrophobic contacts are mediated by VAL188, LEU176, LYS190, and PRO174; van der Waals interactions involve ASN187 and THR175; and THR193 contributes via a Pi-donor hydrogen bond. Lastly, phyllodulcin forms a single hydrogen bond with ASP186; hydrophobic interactions with PRO174, LYS190, and LEU176; and extensive van der Waals contacts with THR175, VAL188, GLU185, TYR181, THR184, HIS171, ASP178, TRP244, and THR193. Collectively, these diverse non-covalent interactions underscore the structural basis for the binding affinities of these polyphenolic compounds to the PPO active site.

CGA (−8.3 kcal mol^−1^) likely bonds perfectly to the catalytic active site (the dicopper center) as a substrate analog. However, hydrangenol (HG, −7.4 kcal mol^−1^), hydrangeic acid (HGA, −7.6 kcal mol^−1^), and phyllodulcin (PD, −7.6 kcal mol^−1^) cannot bind to the dicopper center in the same way because they lack the o-diphenol. Their binding energies likely come from docking to a secondary, allosteric pocket on the enzyme’s surface. Additionally, HG (−7.4 kcal mol^−1^) and HGA (−7.6 kcal mol^−1^) have two separate phenolic -OH groups; PD (−7.6 kcal mol^−1^) has a blocked methoxy and one free -OH. Because they have two free -OH groups at the correct spatial distance, they can bidentately chelate the catalytic copper ions (Cu A and Cu B). PD lacks the two free -OH groups needed for strong copper chelation (it has one -OH and one -OCH_3_) [13]. Therefore, PD barely interacts with the copper at all. Instead, its exceptionally high inhibition rate comes from binding tightly to the protein scaffold (via strong π-π stacking and hydrophobic interactions in an allosteric pocket), which collapses the active site.

## 3. Materials and Methods

### 3.1. Samples

*Hydrangea macrophylla* serrata leaves were provided by Hansabred GmbH Co. KG (Dresden, Germany). Chlorogenic acid (ROTH, Karlsruhe, Germany), hydrangenol (BOC Sciences, Frankfurt am Main, Germany) and phyllodulcin (Abcr GmbH, Karlsruhe, Germany) were purchased for identification.

### 3.2. Extraction and Isolation

#### 3.2.1. Extraction

*Hydrangea macrophylla* serrata leaves were ground at 10,000 rpm for 10 s. Quadruplicate 1 g ± 0.01 g aliquots of the powder were weighed into 50 mL Falcon tubes, mixed with 10 mL of HPLC-grade water, and vortexed for 30 s. The samples were then incubated at 80 °C for 18 h in a vacuum oven (Heraeus, Hanau, Germany) to increase the yield rate of hydrangenol, hydrangeic acid, and phyllodulcin. After fermentation, the mixtures were extracted with 5 mL of methanol and 5 mL of 70% ethanol in an ultrasonic bath for 30 min.

#### 3.2.2. Isolation

Preparative reversed-phase HPLC (Merck KGaA, Darmstadt, Germany and Hitachi Instruments, Inc., San Jose, CA, USA) was performed on a system equipped with a Merck-Hitachi L-7100 pump, L-7400 UV detector, L-5025 column thermostat, and Shimadzu Chromatopac C-R6A integrator (Shimadzu Deutschland GmbH, Duisburg, Germany). Separation was achieved on a C18 column (250 × 8 mm, Nucleodur, Macherey-Nagel, Düren, Germany) maintained at 28 ± 1 °C. The mobile phase, delivered at 4.0 mL/min, consisted of solvent A (0.3% formic acid in HPLC-grade water) and solvent B (acetonitrile). The gradient program was as follows: 70% A for 0–20 min, linearly decreasing to 60% A from 20 to 40 min. With an injection volume of 800 µL, the eluate was monitored by UV absorbance at 254 nm. Target fractions were collected via repeated injections, pooled, and concentrated under reduced pressure (≈10 Pa) at 35 °C using a rotary evaporator. The resulting residues were re-dissolved in methanol, dried under a nitrogen stream, and stored at −25 °C for subsequent analysis. For each subsequent analysis, four samples were extracted as four replicates.

### 3.3. Identification

#### 3.3.1. HPLC-DAD

HPLC-DAD analysis was performed using a system equipped with a Merck-Hitachi L-7100 pump and an L-7450 UV detector (Merck KGaA, Darmstadt, Germany and Hitachi Instruments, Inc., San Jose, CA, USA). Chromatographic separation was achieved on a Luna C18 column (250 × 4.6 mm, 5 μm; Phenomenex, Aschaffenburg, Germany) tempered at 28 ± 1 °C. The mobile phase consisted of solvent A (0.3% formic acid in HPLC-grade water) and solvent B (acetonitrile), delivered at a flow rate of 0.5 mL/min. The linear gradient program was as follows: 0–2 min, 90% A; 2–12 min, linear to 80% A; 12–22 min, linear to 65% A; 22–25 min, hold at 65% A; 25–27 min, linear to 90% A; and 27–37 min, linear to 80% A and held. The injection volume was 20 µL, and the eluate was monitored by UV absorbance at 254 nm.

#### 3.3.2. LC-MS/MS

An HPLC apparatus was connected to an API 4000 QTrap LC-MS/MS system (Applied Biosystems/MDS Sciex, Framingham, MA, USA) equipped with a turbo ion spray source using electrospray ionization in positive mode: a sprayer capillary voltage of 5.5 kV, nebulizing gas flow of 55 mL min^−1^, heating gas flow of 65 mL min^−1^ at 550 °C, and curtain gas flow of 40 mL min^−1^. Chromatographic separations were performed on a stainless-steel column packed with RP-18 material (Vydac 218 TP C18, 250 mm × 4.6 mm, 5 μm; Grace, Columbia, MD, USA) using a flow rate of 1.0 mL min^−1^. The mobile phases used were water (solvent A) and acetonitrile (solvent B). To both solvents (A and B), 0.3% (*v*/*v*) formic acid was added. Analysis was performed at a column temperature of 25 °C using gradient elution: 10% B (2 min) to 20% B (in 10 min) to 35% B (in 10 min, hold for 8 min), to 80% B (in 2 min), held for 5 min. For mass spectrometric detection, the full scan mode (MS1) was used in case of first identification with a declustering potential (DP) of 40 V and an entrance potential of 10 V. Collision-induced dissociation (CID or MS2) of the protonated molecules [M + H]^+^ was achieved by the following parameters: a declustering potential (DP) of 40 V, entrance potential of 10 V, collision energy (CE) of 30 V and cell exit potential (CXP) of 15 V.

### 3.4. Determination of Total Phenolics Content (TPC), ORAC and ABTS˙^+^ Radical Cation Scavenging Activity

The total phenolic content (TPC, Folin–Ciocalteu method, in g gallic acid equivalents L^−1^) and antioxidant activity (ABTS+·, AAPH· in mmol Trolox equivalents L^−1^) were determined as described by Al-Yafeai et al. [1]. Three samples per extraction solution were analyzed. Antioxidant results are expressed on different bases depending on the sample type: g GAE/L for liquid extracts (where the molecular weight of the plant material is unclear), g GAE/100 g for dry plant material (to facilitate comparison with literature values), and mmol TE/L or mM TE/mM for Trolox-equivalent antioxidant capacity assays. The conversion from mmol TE/L to mM TE/mM was calculated by dividing by the molar concentration of the tested compound or the total phenolic concentration of the extract.

### 3.5. Anti-Browning Activity

#### 3.5.1. Extraction and Partial Purification of PPO

PPO in untreated fresh-cut apple fruit was extracted following a recently used method with slight modification [1]. Apples were washed, peeled and cut into 5 mm slices. A 2 ± 0.02 g apple sample was homogenized in 6 mL of phosphate buffer (disodium hydrogen phosphate dihydrate and sodium dihydrogen phosphate monohydrate, pH 6.5) with 5% PvPP (polyvinylpolypyrrolidone, Sigma-Aldrich, Taufkirchen, Germany) for 3 min in an ice bath. Centrifugation at 8000 rpm was done at 4 °C for 30 min, and the resulting supernatant was carefully collected and additionally centrifuged for 10 min at 4 °C and 8000 rpm to collect the aqueous phase.

#### 3.5.2. Enzyme Activity Test

Polyphenol oxidase activity was determined following the method with 4-methylcatechol as the substrate [1]. Upon oxidation, the substrate generates an orange-colored chromophore. Briefly, the enzymatic reaction of 40 μL crude enzyme extract and 120 μL phosphate buffer (50 mM, 33 mM 4-methylcatechol, pH 6.5) was measured for absorbance changes at 420 nm over time at 25 °C. This process was carried out on a BMG LABTECH FLUOstar OPTIMA plate reader using OPTIMA software version 2.20R2 (BMG LABTECH, Ortenberg, Germany). The kinetic protocol comprised 50 cycles (total duration: 32 min), and within each 34 s cycle, the plate was subjected to 1 s of double orbital shaking at 120 rpm directly before the reading. All assessments were conducted in four replicates. Data were analyzed using the Optima Data Analysis program (BMG LABTECH). An increase in absorbance of 0.001 was considered as 1 unit (U) of enzyme activity. The substrate turnover rate (U/(min*g)) was determined by the slope of the absorption curve within the linear phase of the reaction. The linear phase began a few seconds after addition of the substrate and lasted for a brief period before transitioning into a nonlinear phase.

#### 3.5.3. Inhibition and Recovery of Polyphenol Oxidase

The crude PPO extract was diluted in phosphate buffer (pH 6.5). The well compositions were as follows: (A) test wells contained 40 µL of PPO and 120 µL of substrate buffer; (B) blank wells for A contained 160 µL of buffer without substrate; (C) test wells contained 40 µL of inhibitor, 40 µL of PPO, and 80 µL of substrate buffer; (D) blank wells for C contained 120 µL of buffer and 40 µL of inhibitor. The plate was shaken for 5 min and then incubated at 25 °C for 5 min. After the addition of 4-methylcatechol, the absorbance was measured at 420 nm. 4-Methylcatechol exhibits higher catalytic efficiency and tighter enzyme binding compared to unsubstituted catechol. The percentage of *PPO inhibition* was calculated using:

%PPO inhibition=[(((A−B)−(C−D))/(A−B))×100]

The recovery by Cu^2+^ was performed according to Feng et al. [14]. After the PPO extract and inhibitors were incubated for one hour, CuSO_4_ solution was added to the system; the experiment was performed at 25 °C, pH 6.5 for 15 min, and the absorbance was measured at 420 nm.

Recovery rate (%) = [(Arec − Ainh)/(Actrl − Ainh)] × 100

Here, Arec is the activity after Cu^2+^ addition, Ainh is the activity in the presence of inhibitor, and Actrl is the activity of the uninhibited control.

#### 3.5.4. Polyphenol Oxidase Kinetics

IC_50_ values were calculated by nonlinear regression using a four-parameter logistic equation (variable slope) in R studio 4.4.2 [15], based on 0.125–2 mg/mL of each inhibitor. Results are reported with 95% confidence intervals. IC_50_ values are expressed as apparent IC_50_ values based on the measured absorbance response at 420 nm. The kinetic parameters Km and Vmax for polyphenol oxidase were determined by measuring initial reaction velocities at increasing substrate concentrations (pyrocatechol for PPO). Reaction rates were calculated based on the slope of absorbance over time in the linear range. The data were then fitted to the Michaelis–Menten equation using nonlinear regression analysis. The best-fit values of Km and Vmax were obtained from the regression curves, and results are expressed as the mean ± standard deviation from three independent experiments [16]. Kinetic parameters (Km, Vmax) were estimated by nonlinear least squares fitting according to the Michaelis–Menten equation. The results are expressed as Δabs min^−1^ [17]. The inhibition constants for binding to free enzyme (Kis) and to the enzyme–substrate complex (Kii) were determined from Lineweaver–Burk secondary plots. The slope of the primary plot versus the inhibitor concentration gave −Kis, and the intercept versus the inhibitor concentration yielded −Kii.

### 3.6. Molecular Docking

To investigate the binding mode of the four compounds with tyrosinase, a molecular docking study was performed using Autodock Vina 1.1.2 (The Scripps Research Institute). The three-dimensional (3D) coordinates of tyrosinase (PDB ID: 2Y9X) were downloaded from RCSB Protein Data Bank (www.rcsb.org), and 2D models of CGA (ID: 1794427), HG (ID: 119199), HGA (ID: 5318116) and PD (146694) were downloaded from Pubchem (https://pubchem.ncbi.nlm.nih.gov/). The catalytic copper ions (CuA and CuB) were removed during preparation. The blind docking and best-scoring pose were carried out using the Vina docking score and visually analyzed using PyMoL 2.6 software (www.pymol.org, accessed on 28 August 2026). The pose exhibiting the lowest binding energy was selected as the optimal docking solution, representing the most favorable predicted interaction of PPO with CGA, HG, HGA or PD.

### 3.7. Statistics

The data analysis in this study was conducted using R studio 4.4.2 [15]. All experimental analyses were performed in quadruplicate, and the results are presented as the mean ± standard deviation (SD). To compare data from different analyses, a one-way ANOVA (analysis of variance) followed by the Tukey test was employed, enabling the identification of significant differences (*p* < 0.05) among multiple groups. Significant differences (*p* < 0.05) between two groups were identified by paired T-test. In the paired T-test, each pair consisted of two measurements derived from the same replicate, which ensured that the paired observations were naturally matched. Correlations between variables were assessed using Spearman’s correlation coefficient (R). Spearman’s correlation analysis was performed to investigate the relationships between phytochemical contents and antioxidant parameters. A heatmap was generated to visually illustrate the correlation matrix.

## 4. Conclusions

This study comparatively assessed CGA, HG, HGA, and PD for their antioxidant activity and apparent PPO inhibition alongside whole tea extract. The whole tea extract showed higher antioxidant activity than any individual compound, whereas PD (IC_50_ = 0.352 mg/mL) showed stronger apparent PPO inhibition than the whole-leaf extract (IC_50_ = 0.451 mg/mL), indicating that antioxidant capacity and PPO inhibition are not driven by the same constituents and that the extract matrix influences the net anti-browning response. Several limitations should nevertheless be acknowledged: detailed information on the geographical origin, cultivation conditions, harvest period, and botanical authentication of the plant material was not available; blind docking was performed without targeting the catalytic dicopper center (CuA/CuB), so the proposed binding modes remain hypotheses requiring validation through targeted docking or molecular dynamics simulations; and direct comparison with approved anti-browning agents was not possible because of differences in assay conditions, meaning that further testing in real food systems is needed. Future work should therefore obtain complete plant material information, clarify whether the compounds interact directly with CuA/CuB, and evaluate efficacy in actual fruit and vegetable systems. Overall, these results suggest that *Hydrangea macrophylla* subsp. serrata is a promising natural source for mitigating enzymatic browning, pending further validation.

## Figures and Tables

**Figure 1:**
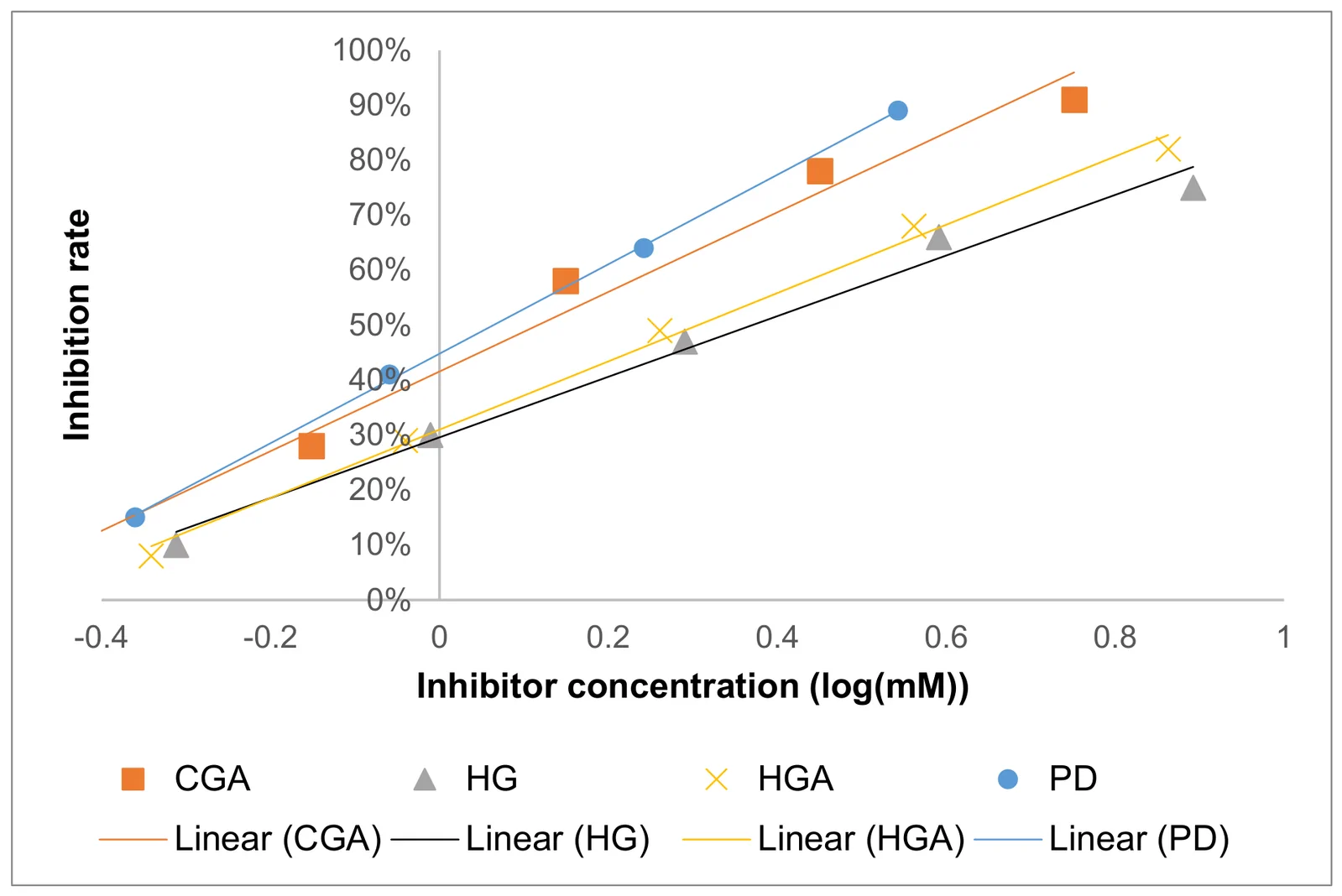
Concentration-dependent inhibition by CGA, HG, HGA, and PD with increasing inhibitor concentrations. CGA: chlorogenic acid; HG: hydrangenol; HGA: hydrangeic acid; PD: phyllodulcin.

**Figure 2:**
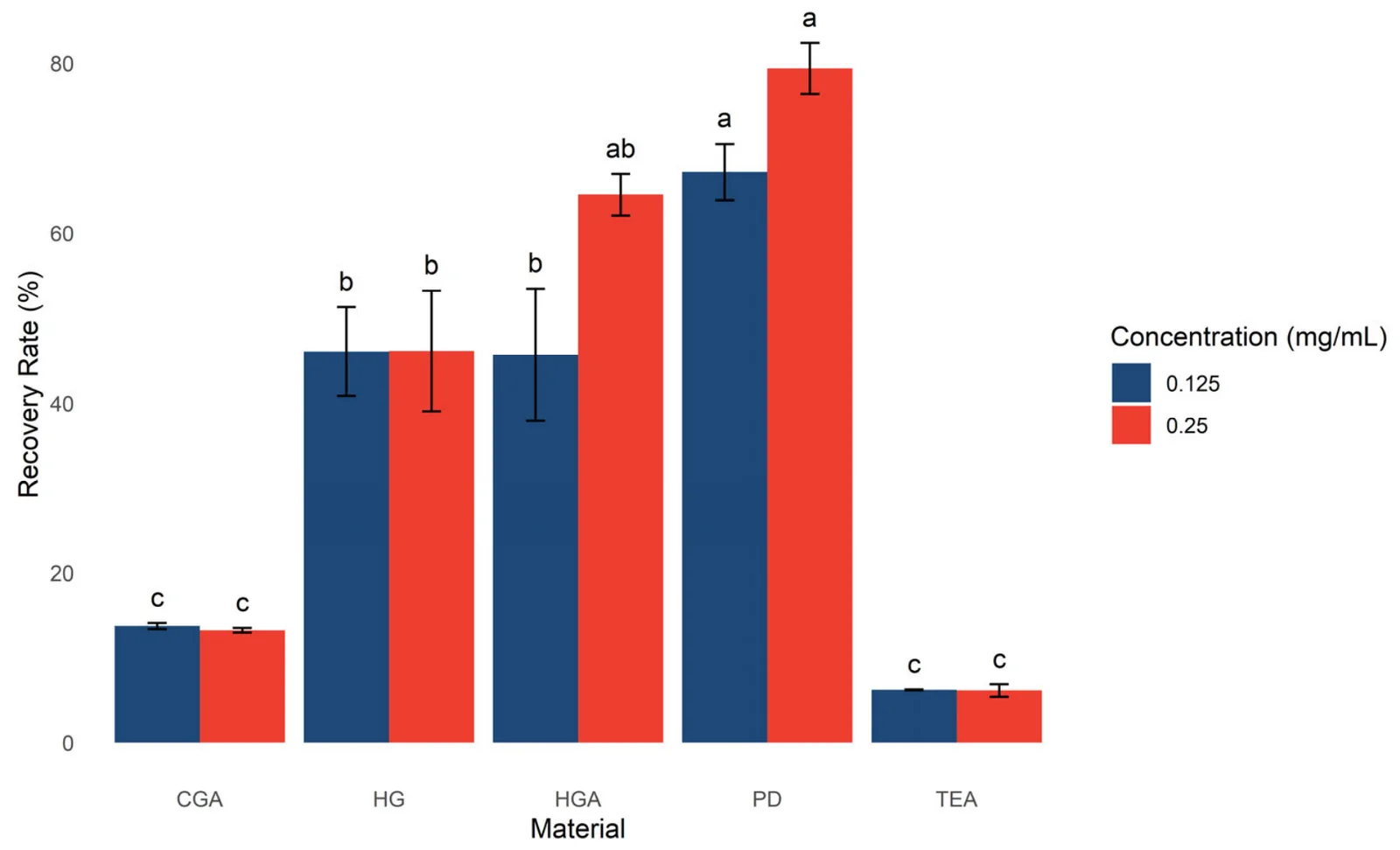
Recovery rate of PPO activity under different inhibitors at 0.125 and 0.25 mg/mL. CGA: cholorogenic acid; HG: hydrangenol; HGA: hydrangeic acid; PD: phyllodulcin. Significance of differences between samples (*p* < 0.05) was determined using one-way ANOVA followed by Tukey test, with different letters (a/b/c) showing a significant difference.

**Figure 3:**
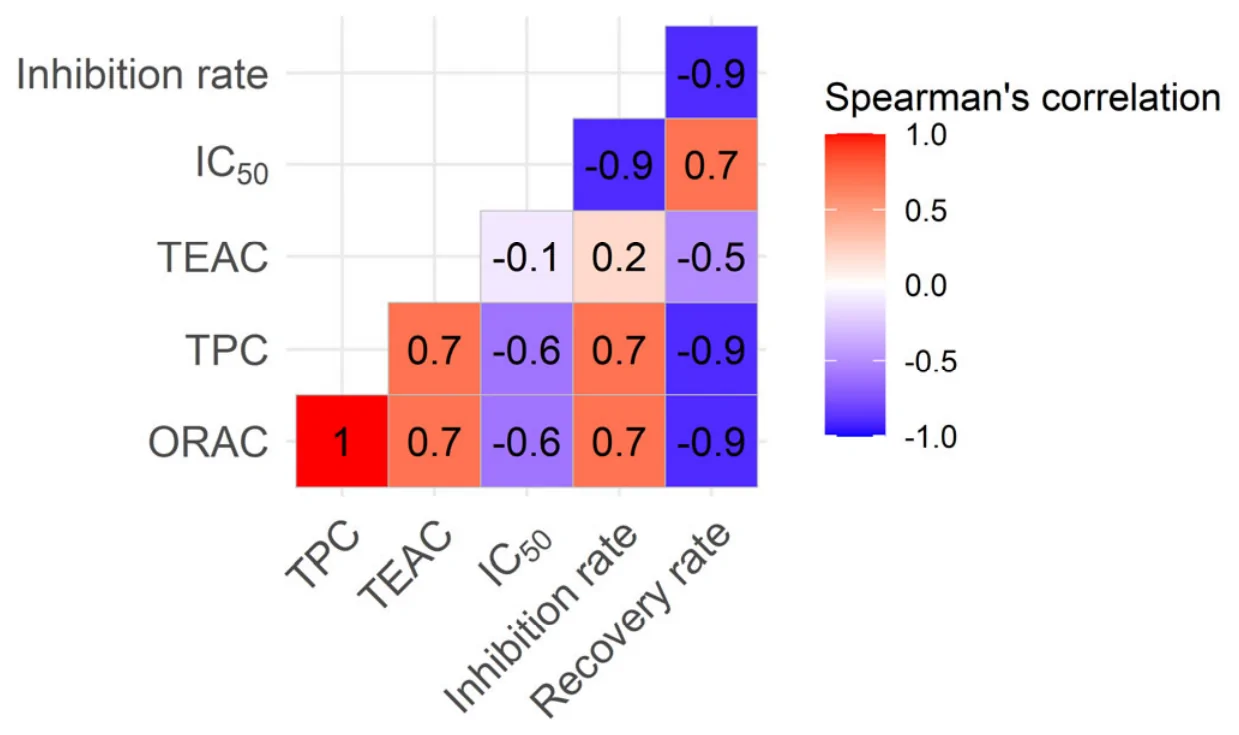
Spearman’s correlation matrix between antioxidant activity and anti-browning activity for the four compounds.

**Figure 4:**
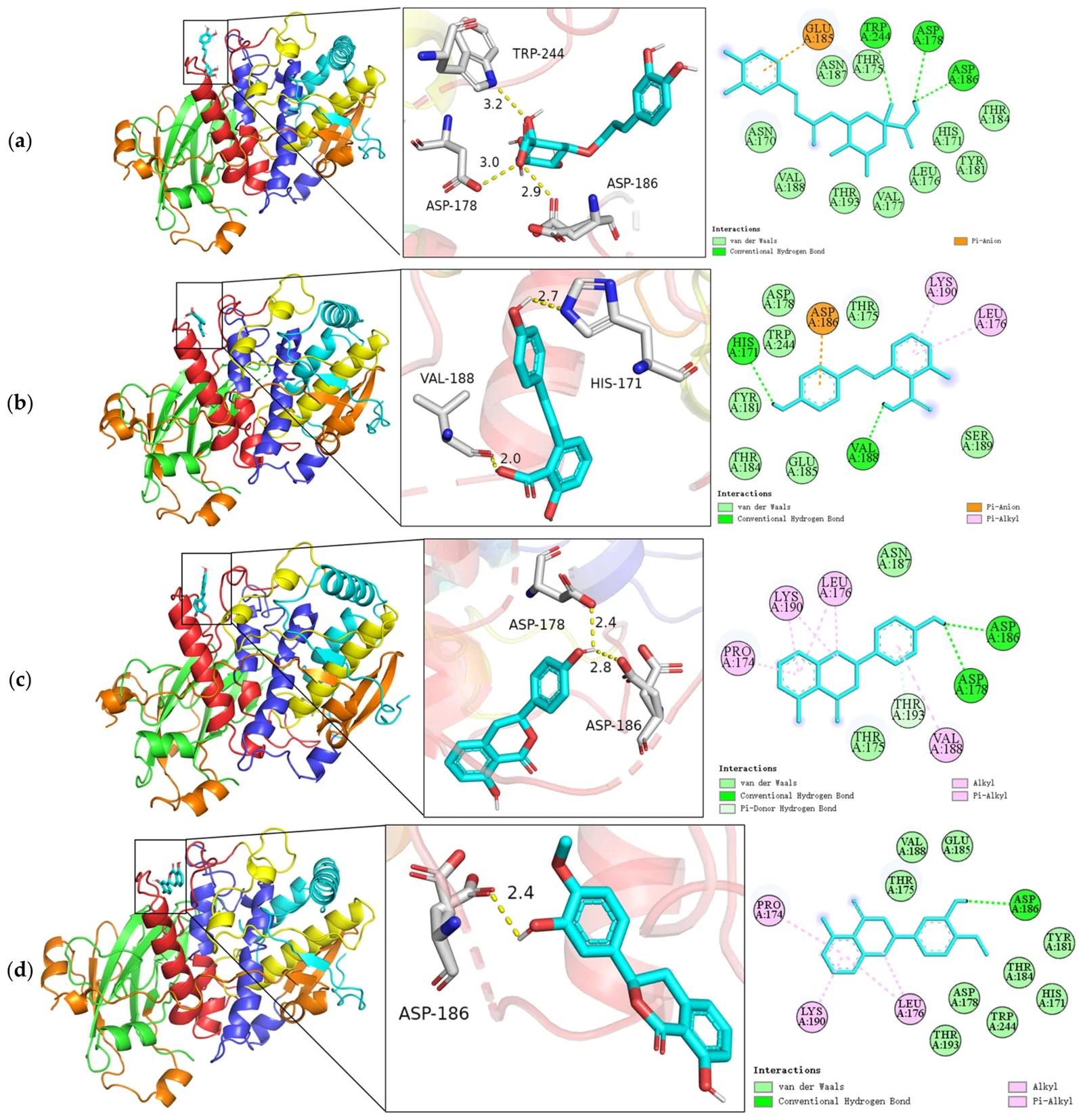
Three-dimensional views and binding models of (**a**) chlorogenic acid, (**b**) hydrangenol, (**c**) hydrangeic acid, and (**d**) phyllodulcin with PPO.

**Table 1:** Antioxidant activities of four compounds via total phenolics content (TPC), Trolox equivalent antioxidant capacity (TEAC), and oxygen radical absorbance capacity (ORAC).

	TPC [g GAE/100 g]	TEAC [mM TE/mM]	ORAC [mM TE/mM]
CGA	3.69 ± 0.16 a	0.20 ± 0.01 a	2.45 ± 0.04 a
HG	0.75 ± 0.09 d	0.08 ± 0.01 b	1.41 ± 0.05 c
HGA	1.12 ± 0.05 c	0.13 ± 0.01 a	2.00 ± 0.04 b
PD	1.77 ± 0.05 b	0.15 ± 0.02 a	2.18 ± 0.04 b

The data are presented as the mean ± standard deviation (SD) for four replicates (n = 4). Significance of differences between samples (*p* < 0.05) was determined using one-way ANOVA followed by the Tukey test, with different letters (a/b/c/d) within a column indicating statistically significant distinctions. TPC: total phenolics content; TEAC: Trolox equivalent antioxidant capacity; ORAC: oxygen radical absorbance capacity; GAE: gallic acid equivalents; TE: Trolox equivalents; CGA: chlorogenic acid; HG: hydrangenol; HGA: hydrangeic acid; PD: phyllodulcin.

**Table 2:** Comparing the antioxidant activities of four compounds and tea extract via total phenolics content (TPC), Trolox equivalent antioxidant capacity (TEAC), and oxygen radical absorbance capacity (ORAC).

	TPC [g GAE/100 g]	TEAC [mmol TE/g]	ORAC [mmol TE/g]
CGA	3.69 ± 0.16 a	0.56 ± 0.02 a	6.92 ± 0.10 a
HG	0.75 ± 0.09 b	0.30 ± 0.01 b	3.98 ± 0.15 b
HGA	1.12 ± 0.05 c	0.49 ± 0.02 a	5.65 ± 0.12 c
PD	1.77 ± 0.05 d	0.54 ± 0.01 a	6.19 ± 0.12 c
Tea	3.95 ± 0.09 e	1.09 ± 0.03 c	12.26 ± 0.42 d

The data are presented as the mean ± standard deviation (SD) for four replicates (n = 4). Significance of differences between samples (*p* < 0.05) was determined using one-way ANOVA followed by the Tukey test, with different letters (a/b/c/d/e) within a column indicating statistically significant distinctions. TPC: total phenolics content; TEAC: Trolox equivalent antioxidant capacity; ORAC: oxygen radical absorbance capacity; GAE: gallic acid equivalents; TE: Trolox equivalents; CGA: cholorogenic acid; HG: hydrangenol; HGA: hydrangeic acid; PD: phyllodulcin.

**Table 3:** Polyphenol oxidase inhibition kinetics of chlorogenic acid, hydrangenol, hydrangeic acid, and phyllodulcin.

	Conc. (mM)	Km (mM)	Vmax (μM/min)	Kis (mM)	Kii (mM)	Inhibition Type
Control		0.047	6.85	-	-	
CGA	0.706	0.311	4.63	0.080	1.47	Mixed
HG	0.976	0.32	6.12	0.147	8.20	Competitive
HGA	0.976	0.349	6.04	0.131	7.28	Competitive
PD	0.873	0.204	5.18	0.184	2.71	Mixed

Km: Michaelis constant; Vamx: maximum velocity; Kis: inhibition constant for slope; Kii: inhibition constant for intercept. CGA: chlorogenic acid; HG: hydrangenol; HGA: hydrangeic acid; PD: phyllodulcin.

## Data Availability

Data are contained within the article and Supplementary Materials.

## Supplementary Materials

The following supporting information can be downloaded at https://www.mdpi.com/article/10.3390/molecules31193411/s1, Figure S1: Mass scan (150–650 *m*/*z*) of the isolated peak of hydrangeic acid showing the quasi-molecular ion (256.9 *m*/*z*) as well as the sodium adduct (279.3 *m*/*z*) and the dimeric sodium adduct 2M+Na (535.3 *m*/*z*); Figure S2: MS2 spectrum of the isolated peak of hydrangeic acid showing the fragmentation pattern resulting from fragmentation of the quasi-molecular ion (256.9 *m*/*z*).

## Abbreviations

The following abbreviations are used in this manuscript:

CGA	Chlorogenic acid
GAE	Gallic acid equivalents
HG	Hydrangenol
HGA	Hydrangeic acid
HPLC-DAD	High-Performance Liquid Chromatography–Diode-Array Detection
LC-MS/MS	Liquid Chromatography–Tandem Mass Spectrometry
ORAC	Oxygen radical absorbance capacity
PD	Phyllodulcin
TE	Trolox equivalents
TEAC	Trolox equivalent antioxidant capacity
TPC	Total phenolics content

## References

[1] Al-Yafeai A., Schmitt B., Malarski A., Böhm V. (2024). In-Vitro Bioactivity Evaluation of Hydrangenol Extracted from Hydrangea Macrophylla (Thunb.) Ser. Leaves. Int. J. Second. Metab..

[2] Kim S.-Y., Chung K.-S., Jang S.-Y., Han H.-S., Heo S.-W., Kil Lee J., Kim H.J., Shin Y.-K., Ahn H.S., Lee S.H. (2023). Hydrangenol, an Active Constituent of Hydrangea Serrata (Thunb.) Ser., Ameliorates Colitis through Suppression of Macrophage-Mediated Inflammation in Dextran Sulfate Sodium-Treated Mice. Food Funct..

[3] Yagi A., Washida Y., Takata N., Nishioka I. (1972). Studies on Hydrangea Species. I. Phenolic Components of Hydrangea Serrata SERINGE Var. Thunbergii SUGIMOTO. Chem. Pharm. Bull..

[4] Yoshikawa M., Matsuda H., Shimoda H., Shimada H., Harada E., Naitoh Y., Miki A., Yamahara J., Murakami N. (1996). Development of Bioactive Functions in Hydrangeae Dulcis Folium. V. On the Antiallergic and Antimicrobial Principles of Hydrangeae Dulcis Folium. (2). Thunberginols C, D, and E, Thunberginol G 3’-O-Glucoside, (-)-Hydrangenol 4’-O-Glucoside, and (+)-Hydrangenol 4’-O-Glucoside. Chem. Pharm. Bull..

[5] Zhang H., Matsuda H., Yamashita C., Nakamura S., Yoshikawa M. (2009). Hydrangeic Acid from the Processed Leaves of Hydrangea Macrophylla Var. Thunbergii as a New Type of Anti-Diabetic Compound. Eur. J. Pharmacol..

[6] Muñoz J., Garcia-Molina F., Varon R., Rodriguez-Lopez J.N., García-Ruiz P.A., García-Cánovas F., Tudela J. (2007). Kinetic Characterization of the Oxidation of Chlorogenic Acid by Polyphenol Oxidase and Peroxidase. Characteristics of the o-Quinone. J. Agric. Food Chem..

[7] Kim E., Shin J.-H., Seok P.R., Kim M.-S., Yoo S.-H., Kim Y. (2018). Phyllodulcin, a Natural Functional Sweetener, Improves Diabetic Metabolic Changes by Regulating Hepatic Lipogenesis, Inflammation, Oxidative Stress, Fibrosis, and Gluconeogenesis in Db/Db Mice. J. Funct. Foods.

[8] Lim W.Y., Wong C.W. (2018). Inhibitory Effect of Chemical and Natural Anti-Browning Agents on Polyphenol Oxidase from Ginger (Zingiber Officinale Roscoe). J. Food Sci. Technol..

[9] Chang X., Wang Q., Dong T., Feng Y. (2025). The Role of Chicory Furaneol in Reducing Potato Browning: Inhibiting Polyphenol Oxidase Activity and Enhancing Antioxidant Capacity. Postharvest Biol. Technol..

[10] Prior R.L., Wu X., Schaich K. (2005). Standardized Methods for the Determination of Antioxidant Capacity and Phenolics in Foods and Dietary Supplements. J. Agric. Food Chem..

[11] Ge C., Zhao X., Cui G., Jiang X., Liu P., Zheng X., Wang C., Xu Y., Qu X., Cao P. (2025). The Natural Product 3-Mercaptopropionic Acid Is a Highly Effective Browning Inhibitor by Directly Capturing Polyphenol Oxidase Activity. LWT.

[12] Fang X., Han J., Lou X., Lv Y., Zhang Y., Xu X., Lv Z., Lu G. (2023). Effect and Mode of Different Concentrations of Citrus Peel Extract Treatment on Browning of Fresh-Cut Sweetpotato. Foods.

[13] Nerya O., Musa R., Khatib S., Tamir S., Vaya J. (2004). Chalcones as Potent Tyrosinase Inhibitors: The Effect of Hydroxyl Positions and Numbers. Phytochemistry.

[14] Feng Y., Liu Q., Liu P., Shi J., Wang Q. (2020). Aspartic Acid Can Effectively Prevent the Enzymatic Browning of Potato by Regulating the Generation and Transformation of Brown Product. Postharvest Biol. Technol..

[15] R Core Team (2025). R: A Language and Environment for Statistical Computing.

[16] Tighilet K., Adjebli A., Messis A. (2025). Inhibition of Enzymatic Browning and Antioxidant Activities of Marrubium Vulgare L Extracts: A Promising Natural Solution. Pharmacol. Res. Mod. Chin. Med..

[17] Lim W.Y., Cheun C.F., Wong C.W. (2019). Inhibition of Enzymatic Browning in Sweet Potato (*Ipomoea batatas* (L.)) with Chemical and Natural Anti-Browning Agents. J. Food Process. Preserv..

