# Supplementary material for: Mechanistic Insights into Polyphenol Oxidase Inhibition by Four Bioactive Constituents from Hydrangea macrophylla Serrata: Antioxidant Activity of Single Compounds Compared to the Whole-Leaf Extract
Source: Molecules. 2026 Sep 24;31(19):3411. doi: 10.3390/molecules31193411 (PMC13666256; doi:10.3390/molecules31193411)
Supplement: Supplementary file 1 [file molecules-31-03411-s001.zip › Mei et al Molecules Supplementary Material Text for Figures S1_S2.pdf]

Mei et al., Molecules 2026 (molecules-4560838)

Supplementary Material

Figure S1. Mass scan (150-650 m/z) of the isolated peak of hydrangeic acid showing the quasi-molecular ion (256.9 m/z) as well as the sodium adduct (279.3 m/z) and the dimeric sodium adduct 2M+Na (535.3 m/z).

Figure S2. MS2 spectrum of the isolated peak of hydrangeic acid showing the fragmentation pattern resulting from fragmentation of the quasi-molecular ion (256.9 m/z).
